# Poster Session II - A270 REAL-WORLD EFFECTIVENESS OF ABP-654 IN PATIENTS WITH INFLAMATORY BOWEL DIESEASE IN CANADA

**DOI:** 10.1093/jcag/gwaf042.270

**Published:** 2026-02-13

**Authors:** A E Servin, M Chohan, M Jayakumarhtt, F Dawod, O Mooney, R Lukanova

**Affiliations:** Center for Observational Research, AMGEN, Thousand Oaks, CA; Center for Observational Research, AMGEN, Thousand Oaks, CA; Center for Observational Research, AMGEN, Thousand Oaks, CA; Adelphi Group Limited, Bollington, England, United Kingdom; Adelphi Group Limited, Bollington, England, United Kingdom; Adelphi Group Limited, Bollington, England, United Kingdom

## Abstract

**Background:**

ABP-654 (WEZLANA®), a biosimilar to ustekinumab (UST), was approved by Health Canada in December 2023 for several immune diseases, including Crohn’s disease (CD) and ulcerative colitis (UC). Real-world data on its use remains limited.

**Aims:**

To provide initial findings on the real-world use and effectiveness of ABP-654 as the first UST biosimilar approved for use in patients with inflammatory bowel disease (IBD) across Canada

**Methods:**

Data were drawn from the Adelphi IBD Disease Specific Programmen ^TM^, a cross-sectional survey with retrospective data collection from physicians conducted in Canada from May-October 2025. Gastroenterologists (GIs) provided data on their patients with IBD receiving ABP-654, who had received >2 subcutaneous doses, including demographics, clinical characteristics, treatment utilization, and satisfaction. Analyses were descriptive.

**Results:**

Seven GIs reported data on 32 patients (15 CD, 17 UC). Mean [standard deviation; SD] age was 46.3 [15.7] years and 59% of patients were male. Mean [SD] time since initiating ABP-654 therapy at the time of consultation was 8.4 [2.5] months; 29 initiated ABP-654 as first UST therapy and 3 previously received UST. At initiation of ABP-654, 94% (30/32) of patients had moderate to severe disease severity and 84% (27/32) of patients’ disease progression was deteriorating, as reported by their GI. Following ABP-654 therapy at the time of consultation, 22% (7/32) of patients had moderate to severe disease severity, and 3% (1/32) of patients’ disease progression was deteriorating (Figure 1A; Table 1). Additionally, 84% (27/32), 69% (22/32), and 84% (27/32) of patients had achieved clinical, endoscopic, and biochemical remission, respectively (Figure 1B). GIs reported they were satisfied and believed this was the best control that could be achieved for 88% (28/32) of patients (Table 1).

**Conclusions:**

After ∼ 8 months of follow-up, findings from this analysis indicate that most patients receiving ABP-654, improved to mild disease, achieved remission outcomes, and GIs reported high satisfaction.

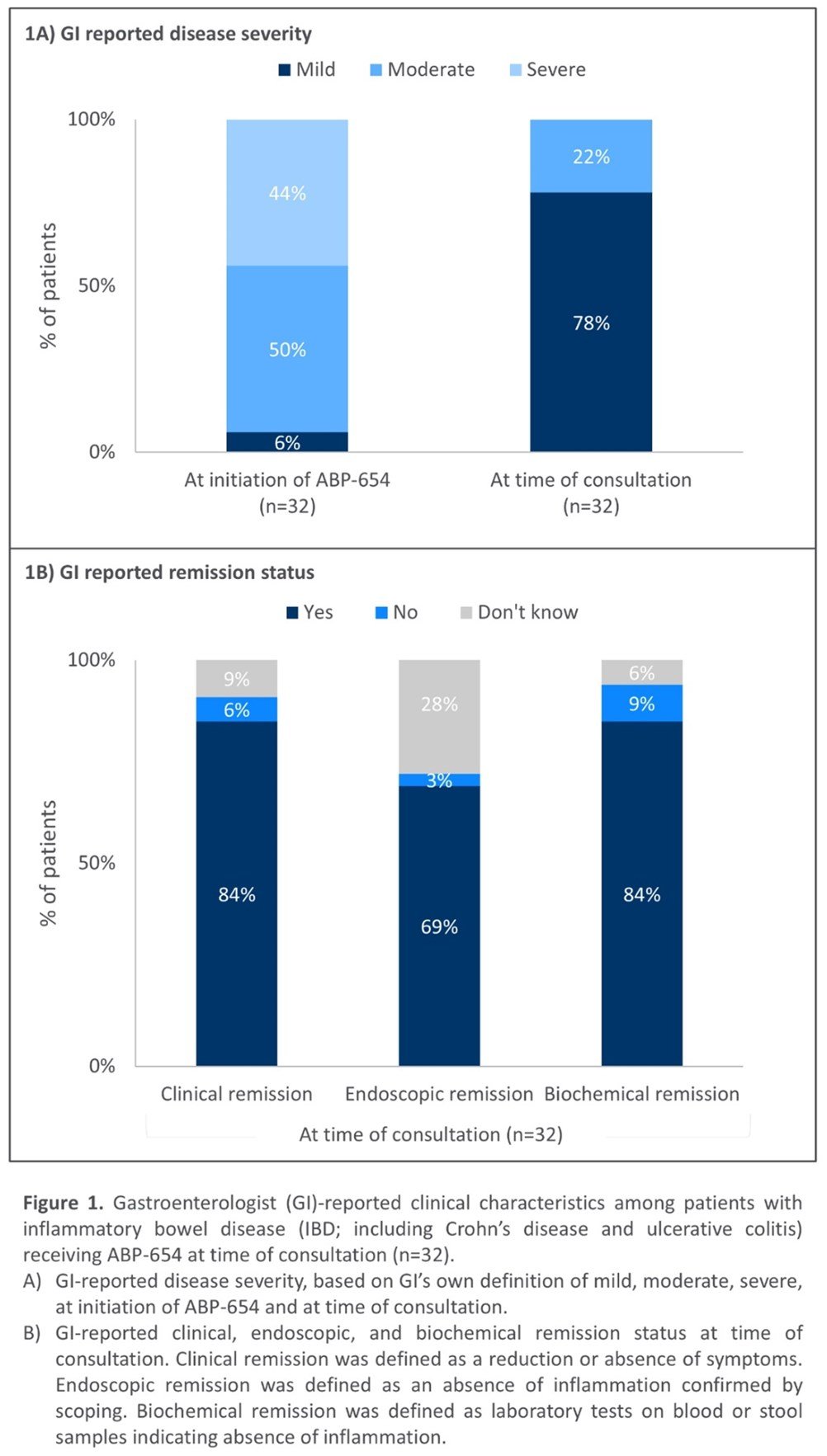

**Funding Agencies:**

Amgen Inc

**A270 Table 1:** Demographics, treatment satisfaction and disease progression of patients with IBD receiving ABP-654

	Patients with IBD receiving ABP- 654
Age, years, mean (SD)	46.3 (15.7)
Sex, male, n (%)	19 (59)
Ethnicity, white, n (%)	23 (72)
Disease duration, years, median [IQR]	3.9 [1.4, 7.3]
GI reported treatment satisfaction	
Satisfied and I believe this is the best control that can be achieved	28 (88)
Satisfied, but I believe better control can be achieved	4 (13)
Not satisfied1	0 (0)
GI reported disease progression at initiation of ABP-654, n (%)	
Improving	1 (3)
Stable	4 (13)
Deteriorating	27 (84)
GI reported disease progression at most recent consultation, n (%)	
Improving	15 (47)
Stable	16 (50)
Deteriorating	1 (3)

IBD, inflammatory bowel disease; SD, standard deviation; IQR, interquartile range; 5-ASA, 5-aminosalicyclate; GI, gastroenterologist *Patients with available data. 1GI reported treatment satisfaction is reported as a four-response categorical outcome. Not satisfied is defined as ‘not satisfied, but I believe this is the best control that can be achieved’, or ‘not satisfied and I believe better control can be achieved’.

